# “*No Ink on Tumor*” in Breast-Conserving Surgery after Neoadjuvant Chemotherapy

**DOI:** 10.3390/jpm12071031

**Published:** 2022-06-23

**Authors:** Giulia Atzori, Marco Gipponi, Chiara Cornacchia, Raquel Diaz, Marco Sparavigna, Maurizio Gallo, Tommaso Ruelle, Federica Murelli, Simonetta Franchelli, Francesca Depaoli, Daniele Friedman, Piero Fregatti

**Affiliations:** 1 Breast Surgery Clinic, San Martino Policlinic Hospital, 16132 Genoa, Italy; giulia.atzori90@libero.it (G.A.); cornacchia.chiara@gmail.com (C.C.); marcosparavigna@gmail.com (M.S.); federica.murelli@unige.it (F.M.); simonettafranchelli@yahoo.it (S.F.); francesca.depaoli@hsanmartino.it (F.D.); friedman@unige.it (D.F.); pfreg@hotmail.it (P.F.); 2 Department Surgical Sciences and Integrated Diagnostic (DISC), School of Medicine, University of Genoa, 16132 Genoa, Italy; raqueldiaz.ge@gmail.com (R.D.); tommirue@gmail.com (T.R.); 3 Department of Internal Medicine (Di.M.I.), University of Genoa, 16132 Genoa, Italy; maurizio.gallo@unige.it

**Keywords:** breast cancer surgery, neoadjuvant chemotherapy, excision

## Abstract

*Background/Aim:* Patients with Stage I-II breast cancer undergoing breast-conserving surgery after neoadjuvant chemotherapy (BCS-NAC) were retrospectively assessed in order to evaluate the extent of a safe excision margin. *Materials and Methods:* Between 2003 and 2020, 151 patients underwent risk-adapted BCS-NAC; margin involvement was always assessed at definitive histology. Patients with complete pathological response (pCR) were classified as the RX group, whereas those with residual disease and negative margins were stratified as R0 < 1 mm (margin < 1 mm) and R0 > 1 mm (margin > 1 mm). *Results:* Totals of 29 (19.2%), 64 (42.4%), and 58 patients (38.4%) were included in the R0 < 1 mm, R0 > 1 mm, and RX groups, respectively, and 2 patients with margin involvement had a mastectomy. Ten instances of local recurrence (6.6%) occurred, with no statistically significant difference in local recurrence-free survival (LRFS) between the three groups. A statistically significant advantage of disease-free survival (*p* = 0.002) and overall survival (*p* = 0.010) was observed in patients with pCR. *Conclusions:* BCS-NAC was increased, especially in HER-2-positive and triple-negative tumors; risk-adapted BCS should be preferably pursued to highlight the cosmetic benefit of NAC. The similar rate of LRFS in the three groups of patients suggests a shift toward the “*no ink on tumor*” paradigm for patients undergoing BCS-NAC.

## 1. Introduction

The use of neoadjuvant chemotherapy (NAC) is increasing; NAC provides several benefits because it allows for in vivo evaluation of chemosensitivity, enables the resection of tumors that were initially deemed technically inoperable, reduces the extent of axillary surgery, increases the rate of breast-conserving surgery (BCS), and seems to improve disease-free survival (DFS) and overall survival (OS) thanks to the anticipation of a systemic treatment in a disease that may be frequently regarded as systemic from its onset, as suggested by the spectrum view [1,2,3,4,5,6].

However, BCS after NAC (BCS-NAC) is more challenging when compared to primary BCS (P-BCS). First, there is still uncertainty regarding the extent of resection, which may be limited to the residual tumor area (risk-adapted BCS) or extended to the original tumor footprint. Secondly, although breast MRI is regarded as the most accurate imaging technique for the assessment of residual disease following NAC, it may overestimate residual disease, potentially increasing the rate of mastectomy [7]. Finally, with regard the width of the margins of excision, it is not clear as to whether the rule of “*no ink on tumor*” that is well accepted in patients with invasive breast cancer undergoing P-BCS can be translated to BCS-NAC [8,9]. The aim of this retrospective study was to evaluate the optimal margin width after BCS-NAC in patients with Stage I-II breast cancer not amenable to P-BCS by assessing the most relevant oncologic outcomes.

## 2. Materials and Methods

Between 2003 and 2020, a retrospective analysis of 151 patients undergoing BCS-NAC was performed at the Breast Surgery Clinic of the Policlinic San Martino Hospital in Genoa, Italy. Data were extracted from an institutional database including clinical, imaging, and histopathological information. The original tumor area underwent preoperative localization by means of sonographically injected sterile charcoal solution before NAC in order to ease the detection of residual tumor after NAC, especially in patients with clinical (cCR) or pathological complete response (pCR), the latter being defined as absence of residual invasive disease without lymph node metastases. Tumor (T) size was monitored throughout the duration of NAC treatment by means of imaging (breast MRI, N = 103; mammography, N = 9; sonography, N = 22) or clinical assessment (N = 17). The prevalence of breast MRI as compared to the other imaging techniques was due to its higher accuracy for assessment of residual disease after NAC as compared to the other diagnostic tools; clinical assessment was always re-evaluated by means of sonography [10].

Surgical treatment included lumpectomy with sentinel lymph node biopsy (SLNB) or lumpectomy with level I-II axillary lymph node dissection; NAC schedules, as well as post-operative medical and radiation therapy (RT), were also prescribed according to updated national guidelines [8]. The NAC regimen included administration of anthracyclines (FEC90/EC90) + taxanes (Paclitaxel 180) in 78% of patients, whereas 22% of patients were given only anthracyclines. Adjuvant RT included whole-breast irradiation in 103 patients (68.2%) and whole-breast irradiation + tumor bed boost in 48 patients (31.8%).

With regard to surgical T treatment, the policy of risk-adapted BCS was followed in order to highlight the cosmetic outcome of the downstaging effect of NAC; whenever margin involvement was detected at definitive histology, patients underwent mastectomy. The pathological examination was performed according to standard techniques whenever residual disease was detected; conversely, in patients with cPR, the whole pathological specimen underwent serial sectioning [11]. Clinical, imaging, and pathologic parameters included age, tumor histotype, grading, hormonal receptor status, BRCA mutation, subtypes based on tumor biology, pre- and post-operative nodal status, TNM disease stage, type of NAC response (partial response, cCR, or pCR), ipsilateral recurrences, regional and/or systemic relapse, and duration of follow-up.

Patients with residual disease and negative margins were stratified into two groups: margin < 1 mm (R0 < 1 mm) and margin > 1 mm (R0 > 1 mm); moreover, patients with pCR were classified as the RX group. The endpoints of the study included: local recurrence-free survival (LRFS), which was defined as the lap of time from surgery to recurrence in the ipsilateral breast; disease-free survival (DFS); and overall survival (OS). Each patient pre-operatively signed an informed consent regarding both treatment-related information and the scientific purposes of the study.

### Statistical Analysis

Descriptive statistics were computed for all variables. A Pearson chi-square test was used to investigate differences of distributions of categorical variables. Survival curves were estimated using the Kaplan–Meier method and compared using a log-rank test. All reported *p*-values are two-sided. A *p*-value < 0.05 was considered statistically significant. Statistical analysis was performed using SPSS version 20.0 for Windows (SPSS Inc., Chicago, IL, USA). 

## 3. Results

Overall, 151 female patients were included in the study, with a median age of 52.65 years; mean and median follow-up were 51.28 and 46 months, respectively. Eight patients had BRCA mutation. Baseline clinical and pathologic features are reported in Table 1. As for the margin width, there were 29 patients (19.2%) in the R0 < 1 mm group, 64 patients (42.4%) in the R0 > 1 mm group, and 58 patients (38.4%) in the RX group; notably, in eight patients (13.8%) of the RX group, in situ ductal carcinoma (DCIS) was detected at definitive histology, but none of them developed local recurrence.

Our study of the distribution of categorical variables showed that there were no statistically significant differences between the three groups, including the variables related to the chemotherapy and radiation therapy regimen. Involved margins at definitive histology were reported in two patients, both of whom underwent total mastectomy. Local recurrence was detected in 8 patients (5.3%), with distant metastasis in 21 patients (13.9%), and 2 patients (1.3%) had both local and systemic relapse. Lymph node involvement occurred in 61 patients (40%); overall, 15 patients (9.9%) died during follow-up. 

### Oncologic Outcome

**Figure 1:**
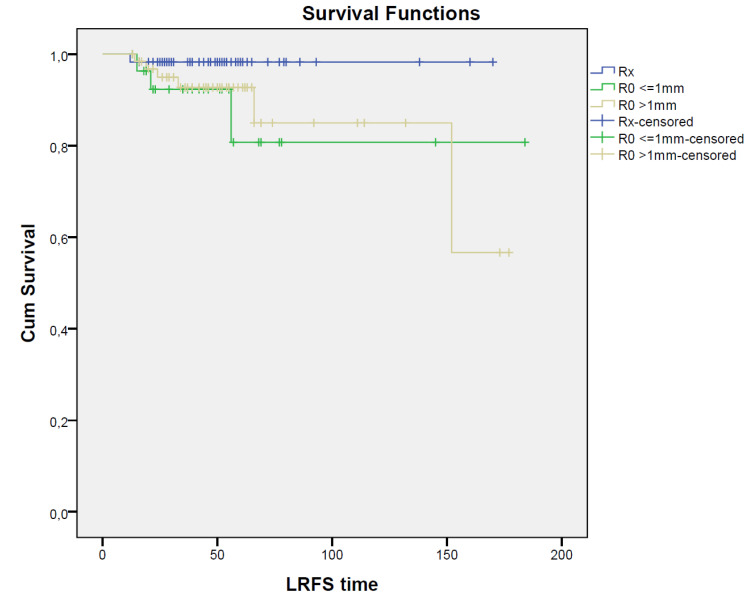

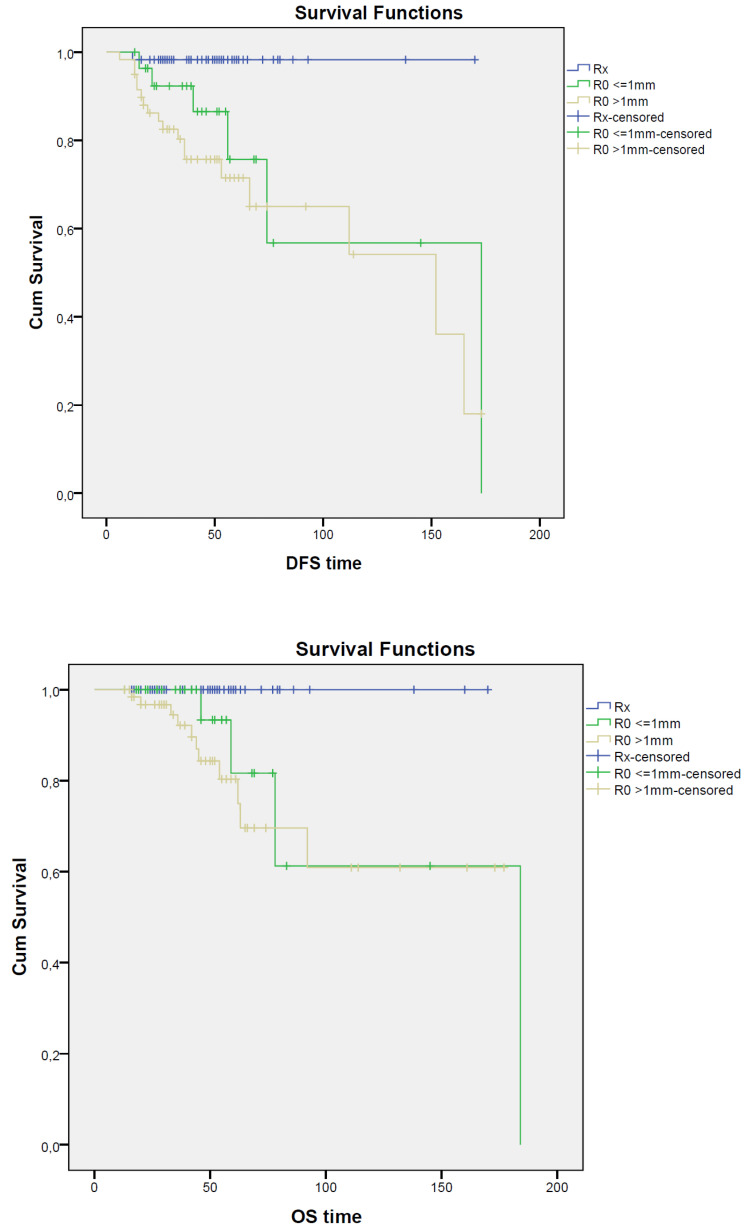
Survival curves for RX, R0 < 1 mm, and R0 > 1 mm groups. Kaplan–Meier curves show LRFS, DFS, and OS for patients with RX (blue solid), ‘R0 < 1 mm (green solid), and ‘R0 > 1 mm (yellow solid) resection; time units: months.

Overall, local recurrence was detected in 10 patients (6.6%), with no statistically significant difference in LRFS in the RX (N = 1; 1.7%), R0 < 1 mm (N = 3; 10.3%), and R0 > 1 mm (N = 6; 9.4%) groups of patients (*p* = 0.177); the corresponding DFS values were 96.6%, 79.3%, and 64.1%, respectively, with a statistically significant advantage in patients who developed a pCR after NAC (*p* = 0.002). Moreover, OS was significantly improved (*p* = 0.010) in patients with pCR; OS was 100% in the RX group, 86.2% in the R0 < 1 mm group, and 82.8% in the R0 > 1 mm group of patients; survival curves are reported in Figure 1.

## 4. Discussion

NAC was initially reserved for locally advanced breast cancer to convert inoperable to operable disease but is now also proposed in early-stage breast cancer [12]. First, the anticipation of a systemic therapy at the beginning of the therapeutic planning was expected to improve the survival rate in patients at risk of distant failure, although the results of a meta-analysis including 4756 breast cancer patients from ten clinical trials performed from 1983 to 2002 undergoing NAC or post-operative adjuvant treatment did not demonstrate any significant advantage either with regard to distant relapse (15-year risk: 38.2% vs. 38%; RR, 1.02; 95% CI, 0.92–1.14; *p* = 0.66), breast-cancer-related mortality (15-year risk: 34.4% vs. 33.7%; RR, 1.06; 95% CI, 0.95–1.18; *p* = 0.31), and overall survival (15-year risk: 40.9% vs. 41.2%; RR, 1.04; 95% CI, 0.94–1.15; *p* = 0.45) unless a pCR is achieved [13]. Secondly, NAC may achieve disease downstaging both at the tumor site and in the axilla in order to increase the rate of BCS, as well as targeted axillary dissection [1]. For instance, in a retrospective 5-year study, Spronk et al. [14] reported an increase in BCS after NAC from 43% to 57%. Thirdly, the extent of response to NAC, namely pCR, has a relevant prognostic role at the individual level, as confirmed by a meta-analysis including 11,955 patients from twelve clinical trials; a significant correlation between pCR and DFS (hazard ratio, 0.48; 95% CI, 0.43–0.54) and OS (hazard ratio, 0.36; 95% CI, 0.31–0.42) was reported in each tumor subtype (luminal A/B, HER2-positive, and triple-negative tumors), although the strength of association was higher for more aggressive subtypes (HER2-positive and triple-negative) [15].

However, a considerable variability regarding the outcome of BCS-NAC can be appreciated, as confirmed in a recent meta-analysis including data from 5379 patients treated with NAC and 10,110 without NAC from 26 studies; the meta-analysis showed wide ranges of tumor-involved margins (2–39.8%), secondary surgeries (0–45.4%), excision volumes (43.2–268 cm^3^), or specimen weight (26.4–233 g) after NAC [16]. This variability may be related to the retrospective nature of these studies, with the inherent heterogeneity and high-risk of bias, but also to the observation that notwithstanding the demonstrated feasibility of BCS, which was increased from 43.3% to 60.4% (*p* < 0.001), BCS was actually performed in 51.8% of patients because only 31% of patients who were eligible for BCS underwent breast conservation (pooled rate ratio, 0.31; 95% CI, 0.22–0.74; *p* = 0.003).

This reduced rate of BCS-NAC may be related to various factors, such as the modality used for the assessment of the tumor response to NAC, with breast RMI being the most reliable imaging technique as compared to other diagnostic modalities, although it could potentially overestimate residual disease, hampering the possibility of a conservative approach [10,17,18]. Another factor might be represented by the pattern of disease response, that is, cCR, pCR: incomplete response of concentric type; or “scattered”: microscopic foci spread over an area similar in size to the original area occupied by the intact tumor [19,20]. Uncertainty may also be related to the extent of resection, which may be limited to the residual tumor (yT) area (risk-adapted BCS) or to the original tumor footprint. Recent data suggest that it is not necessary to excise the entire original tumor volume unless diffuse microcalcification is present; otherwise, the downstaging benefit of NAC would be negated [21,22]. Other predictors of locoregional recurrence are related to the multifocal/multicentric pattern of the original and residual disease; inflammatory breast cancer; presence of lymphovascular invasion into the specimen; tumor subtype, with HER2-positive and triple-negative tumors having the lowest rate of margin positivity because they are more sensitive to chemotherapy as compared to luminal A/B or invasive lobular carcinoma; and gene status and the patient’s preference, in particular the presence of BRCA mutations that may frequently be associated with more extensive and often bilateral procedures [23,24,25,26,27,28,29,30].

In our experience, the width of a safe excision margin was specifically addressed because, dealing with volumes, any undue enlargement of lumpectomy may impair the final cosmetic outcome. For instance, as suggested by Lannin et al. [31], the volume of the operative specimen in the case of a 3–5 cm tumor with a 1 cm excision margin was three to four times higher than the original tumor volume, and the smaller is the tumor, the higher the excessive volume to be resected. Conventionally, in patients with early-stage breast cancer undergoing P-BCS, clear margins of excision are mandatory in order to avoid recurrence due to residual tumor cells (*ipsilateral breast tumor recurrence*, IBTR), and, according to recent guidelines, the standard for invasive breast cancer should be “*no ink on tumor*” [32,33,34]. Conversely, in patients with a diagnosis of ductal carcinoma in situ (DCIS), a 2 mm clearance from the margins of excision is recommended to minimize IBTR, whereas larger resections do not guarantee any added benefit [35,36,37].

In our cohort of patients, analysis of LRFS at a median follow-up of 46 months did not reveal any statistically significant differences between the three groups (RX; R0 < 1 mm; R0 > 1 mm). This finding supports the hypothesis that the distance between the excision margin and the residual tumor has no influence on the development of local recurrence, so the paradigm of “*no ink on tumor*” could also be shifted to patients undergoing BCS-NAC. Due to the low number of local recurrences (N = 10 patients, 6.6%), it was not possible to perform a regression analysis with the aim of identifying risk factors of recurrence, although there was a trend toward a protective effect against recurrence for HER-2-positive and triple-negative tumors, according to literature data [14,16]. Moreover, the statistically significant advantage of DFS and OS in patients with pCR confirms previous observations suggesting that NAC increases survival in this specific subset of patients [14,38,39].

Few other studies have assessed the effect of margin width on prognosis in patients treated with BCS-NAC. Rouzier et al. [40] found that margins <2 mm were associated with increased local recurrence (*p* = 0.04); conversely, other authors reported no associations between LRFS and margin distances [22,41,42,43,44]. A limitation of this and similar studies for the assessment of the width of excision margins is represented by the pattern of tumor regression after NAC (concentric vs. scattered) because this is not often reported in imaging examination, although it represents a fundamental parameter to perform a “*no ink on tumor*” glandular resection, using the yT size as surgical target.

Moreover, uncertainty does exist with respect to the need of an intraoperative diagnosis of margin status, as well as how to manage patients with positive excision margins. As to the former, definitive histopathological examination seems more rewarding because there is a need to properly identify the tumor bed when a cPR does occur or to perform a comprehensive sampling of the circumferential margins to check the margin width when a residual tumor is found [18]. With regard to the management of patients with involved excision margins, even in standard P-BCS, this represents a well-known risk factor for local recurrence, and therefore, additional local therapy is required, such as a RT boost, re-excision, or even mastectomy [45]. Gentilini et al. [46] evaluated the prognostic role of positive excision margins in 198 patients following BCS-NAC: involved margins were found in 21 patients, and four of them underwent reoperation (N = 3 mastectomy; N = 1 re-excision); the other patients had RT only. At 3-year follow-up, the cumulative incidence of local recurrence was 4.7% in patients with negative margins and 13.3% in patients with positive margins (*p* = 0.05), whereas the cumulative incidence of distant metastases was comparable in the two groups (*p* = 0.16), with no significant difference in OS (*p* = 0.577). According to Dutch guidelines, focally involved margins, i.e., a residual tumor in the resection surface over a maximum length of 4 mm, do not require re-excision, whereas in the case of more than focally positive margins, re-excision is mandatory, followed by RT [14]. Conversely, according to American guidelines, whenever there is a “scattered” response and “*if viable tumor is present throughout the specimen even if it does not extend to the margin, a further re-excision should be considered*” [47]. In our experience, no matter the extent of margin involvement, a mastectomy was usually performed unless there was a more than favorable tumor-to-breast volume ratio.

## 5. Conclusions

NAC increases the rate of BCS by downstaging the tumor, especially in HER-2-positive and triple-negative tumors. Similar LRFS rates were observed in patients with pCR, clear margin < 1 mm, and >1 mm, so that the “*no ink on tumor*” paradigm could be safely shifted to patients undergoing BCS-NAC. Risk-adapted BCS should be preferably pursued with the aim of highlighting the benefit of NAC downstaging and the cosmetic outcome, except in the case of diffuse microcalcifications, multifocal/multicentric pattern of disease, or a previous diagnosis of inflammatory breast cancer. The pattern of tumor regression should be accurately investigated prior to BCS-NAC in order to identify patients with “scattered” regression who are at increased risk of positive excision margins. Based on the individual preference, patients with involved margins at definitive histology would preferably undergo mastectomy with immediate breast reconstruction based on adjuvant treatment planning.

## Figures and Tables

**Table 1:** Clinicopathological features and pattern of relapse.

		RX (N = 58)		R0 < 1 mm (N = 29)		R0 > 1 mm (N = 64)		Total (N = 151)	
**Age**	*<50 years*	30	51.7%	16	55.2%	22	34.4%	68	45.0%
	*>50 years*	28	48.3%	13	44.8%	42	65.6%	83	55.0%
**Histotype**	*ductal*	48	82.8%	25	86.2%	59	92.2%	132	87.4%
	*lobular*	1	1.7%	3	10.4%	4	6.2%	8	5.3%
	*other*	9	15.5%	1	3.4%	1	1.6%	11	7.3%
**HR**	*HR+*	27	46.6%	23	79.3%	49	76.6%	99	65.6%
	*HR-*	30	51.7%	6	20.7%	15	23.4%	51	33.8%
	*NA*	1	1.7%	0	0.0%	0	0.0%	1	0.6%
**KI67**	*<20%*	10	17.2%	12	41.4%	22	34.4%	44	29.1%
	*>20%*	48	82.8%	17	58.6%	42	65.6%	107	70.9%
**HER-2**	*positive*	19	32.8%	8	27.6%	14	21.9%	41	27.2%
	*negative*	39	67.2%	20	69.0%	49	76.5%	108	71.5%
	*NA*	0	0.0%	1	3.4%	1	1.6%	2	1.3%
**cN status**	*cN+*	23	39.7%	13	44.8%	29	45.3%	65	43.0%
	*cN0*	35	60.3%	16	55.2%	35	54.7%	86	57.0%
**Lymph node cytology**	*C1*	1	1.7%	0	0.0%	1	1.5%	2	1.3%
	*C2*	6	10.3%	5	17.2%	9	14.1%	20	13.2%
	*C5*	16	27.6%	12	41.4%	21	32.8%	49	32.5%
	*NA*	35	60.4%	12	41.4%	33	51.6%	80	53.0%
**Radiologic response**	*partial*	12	20.7%	22	75.9%	50	78.1%	84	55.6%
	*complete*	46	79.3%	4	13.8%	9	14.1%	59	39.1%
	*no*	0	0.0%	3	10.3%	5	7.8%	8	5.3%
**Pathological response**	*partial*	0	0.0%	26	89.7%	58	90.6%	84	55.6%
	*pCR*	58	100.0%	0	0.0%	1	1.6%	59	39.1%
	*no*	0	0.0%	3	10.3%	5	7.8%	8	5.3%
**Stage**	*0*	58	100.0%	0	0.0%	0	0.0%	58	38.5%
	*1*	0	0.0%	14	48.3%	25	39.0%	39	25.8%
	*2*	0	0.0%	8	27.6%	22	34.4%	30	19.9%
	*3*	0	0.0%	7	24.1%	12	18.8%	19	12.5%
	*4*	0	0.0%	0	0.0%	5	7.8%	5	3.3%
**Subtype**	*luminal A*	0	0.0%	12	41.4%	16	25.0%	28	18.5%
	*luminal B*	10	17.2%	5	17.2%	19	29.7%	34	22.5%
	*HER2+*	19	32.8%	5	17.2%	14	21.9%	38	25.2%
	*triple-negative*	28	48.3%	6	20.7%	14	21.9%	48	31.8%
	*NA*	1	1.7%	1	3.5%	1	1.5%	3	2.0%
**Recurrence**	*local*	1	1.7%	2	6.9%	5	7.8%	8	5.3%
	*systemic*	1	1.7%	3	10.3%	17	26.6%	21	13.9%
	*local-systemic*	0	0.0%	1	0.66%	1	0.66%	2	1.32%
**LR event**	*yes*	1	1.7%	3	10.3%	6	9.4%	10	6.6%
	*no*	57	98.3%	26	89.7%	58	90.6%	141	93.4%
**DFS event**	*yes*	2	3.4%	6	20.7%	23	35.9%	31	20.5%
	*no*	56	96.6%	23	79.3%	41	64.1%	120	79.5%
**OS event**	*yes*	0	0.0%	4	13.8%	11	17.2%	15	9.9%
	*no*	58	100.0%	25	86.2%	53	82.8%	136	90.1%

Legend: NA, not available; HR, hormone receptor status; cN, clinical node; C1, not adequate, C2, benign; C5, cancer; pCR, pathological complete response; LR, local recurrence; DFS; disease-free survival; OS, overall survival.

## Data Availability

Data supporting results are available in Insititutional database.

## Abbreviation

NAC	neoadjuvant chemotherapy
BCS	breast-conserving surgery
PBCS	primary breast-conserving surgery
BCS-NAC	breast-conserving surgery after NAC
cCR	clinical complete response
pCR	pathological complete response
(DCIS)	ductal carcinoma in situ
LRFS	local recurrence-free survival
DFS	Disease-free survival
OS	overall survival
T	tumor size
